# The impact of decreased prognostic nutritional index on the prognosis of patients with pneumonia treated with glucocorticoids: a multicenter retrospective cohort study

**DOI:** 10.3389/fnut.2025.1625531

**Published:** 2025-09-15

**Authors:** Fengwang Xue, Qingmei Fang, Kuangyang Yu, Ruoqing Lu, Xueshuang Chen, Xia Qing, Hong Xiong, Jianhua Peng, Shengmin Guo

**Affiliations:** ^1^School of Nursing, Southwest Medical University, Luzhou, China; ^2^Department of Neurosurgery, the Affiliated Hospital of Southwest Medical University, Luzhou, China; ^3^Department of General Surgery, Luzhou People's Hospital, Luzhou, China; ^4^Department of Nursing, The Affiliated Hospital of Southwest Medical University, Luzhou, China

**Keywords:** prognostic nutritional index, respiratory infections, glucocorticoids, prognosis, all-cause mortality

## Abstract

**Background:**

Long-term or high-dose glucocorticoid administration can markedly impair immune responses, mask clinical indicators of pulmonary infections, and increase the susceptibility to refractory pneumonia, leading to heightened mortality risk. The Prognostic nutritional index (PNI), derived from peripheral lymphocyte count and serum albumin (ALB) levels, serves as a reliable indicator for evaluating nutritional and immune statuses across various clinical populations, including oncology patients, individuals with cardiovascular disorders, and perioperative patients. However, the predictive value of PNI in pneumonia patients receiving glucocorticoids, especially within the Chinese population, has not been sufficiently investigated. This observational analysis aimed to explore the correlation between PNI levels and all-cause mortality (ACM) in patients undergoing prolonged glucocorticoid therapy for pneumonia.

**Methods:**

A retrospective cohort study was conducted utilizing data extracted from the Dryad database. Kaplan–Meier curves, multivariable Cox regression, restricted cubic splines (RCS), and subgroup analyses were used to assess the association between PNI and ACM in patients with pneumonia who received glucocorticoids.

**Results:**

The study incorporated a total of 639 pneumonia patients who received glucocorticoid therapy. The ACM rates were 22.5% at 30 days and rose to 26.0% at 90 days. Multivariable Cox regression showed that, after full adjustment for potential confounders, every 2-unit decrease in PNI was associated with a 10% higher 30-day mortality hazard (*HR* = 1.10, 95% *CI =* 1.05–1.15, *p* < 0.001) and a 9% higher 90-day mortality hazard (*HR* = 1.09, 95% *CI* = 1.04–1.14, *p* < 0.001). Compared with patients with PNI ≥ 43, patients with PNI < 43 had a 118% increased risk of 30-day mortality (*HR* = 2.18, 95% *CI* = 1.28–3.81, *p* = 0.005) and a 96% increased risk of 90-day mortality (*HR* = 1.96, 95% *CI* = 1.20–3.19, *p* = 0.008). Further validation using RCS analysis revealed a robust inverse relationship between PNI scores and ACM, and subgroup analyses revealed no significant interactions.

**Conclusion:**

Among pneumonia patients receiving glucocorticoid therapy, a decreased PNI was associated with an increased risk of 30-day and 90-day mortality, particularly in those with a PNI < 43.

## 1 Background

Pneumonia, a prevalent respiratory disorder, is characterized by inflammation of the terminal airways, alveoli, and interstitium, posing a significant challenge to global health and healthcare systems. Data from 2019 revealed that lower respiratory infections, pneumonia included, ranked as the fourth most frequent cause of global mortality (1), resulting in approximately 2.18 million deaths each year (2). Mortality among pneumonia patients is often heightened by concurrent chronic conditions such as atrial fibrillation, and heart failure (3). The coexistence of these chronic illnesses complicates therapeutic strategies and escalates the mortality risk (4). Additionally, immune suppression resulting from extended or high-dose corticosteroid therapy markedly increases susceptibility to infections, further contributing to elevated pneumonia-related mortality (5, 6). Considering the widespread clinical employment of corticosteroids, it is essential to carefully balance their therapeutic efficacy against possible harmful consequences. Therefore, the timely identification of high-risk patients for poor clinical outcomes is crucial.

Mounting evidence has demonstrated that diminished immune responses and inadequate nutritional status significantly increase susceptibility to infectious agents. Thus, timely and accurate evaluation of nutritional and immunological status is essential for prognostic prediction (7, 8). In China, nutritional interventions are increasingly prioritized by healthcare providers, prompting nursing associations across various administrative levels to develop specialized nutritional training for nurses. Currently, the Nutrition Risk Screening 2002 is the only validated assessment method with demonstrated high accuracy and sensitivity (9, 10). However, the method’s practicality is limited in patients with altered consciousness or large pleural effusions, and the results may be influenced by evaluator subjectivity. Hence, there is an urgent need to develop a simplified, objective, and universally applicable tool to accurately assess the nutritional and immune statuses of pneumonia patients receiving glucocorticoid treatment.

The Prognostic nutritional index (PNI), derived from peripheral lymphocyte count and serum albumin (ALB) concentrations, has been clinically validated as an objective indicator for evaluating nutritional and immune conditions (11). Initially introduced by Onodera et al. (12), the PNI has been broadly adopted across numerous clinical settings, such as oncology (13), cardiovascular diseases (8), chronic kidney disease (14), and community-acquired pneumonia (CAP) (15), demonstrating predictive capabilities regarding therapeutic effectiveness and surgical outcomes. However, there remains limited research specifically examining the association between PNI levels and clinical prognosis in pneumonia patients undergoing glucocorticoid therapy, particularly within Chinese patient populations. Therefore, this study utilized publicly accessible data from the Dryad database to investigate the correlation between PNI and clinical outcomes in this particular patient demographic.

## 2 Methods

### 2.1 Data source

The source data were acquired from the DATA-DRYAD repository,^1^ originally published by Li et al. (5). Ethical approval for the original dataset was granted by the Ethics Committee of the China-Japan Friendship Hospital (Approval No.: 2015–86). This dataset is openly accessible under the Creative Commons Attribution Non-Commercial (CC BY-NC 4.0) license, allowing for non-commercial usage contingent upon proper acknowledgment of the original authors and the data source. The dataset’s reliability has previously been validated by two separate independent studies (6, 16).

### 2.2 Study population

**Figure 1:**
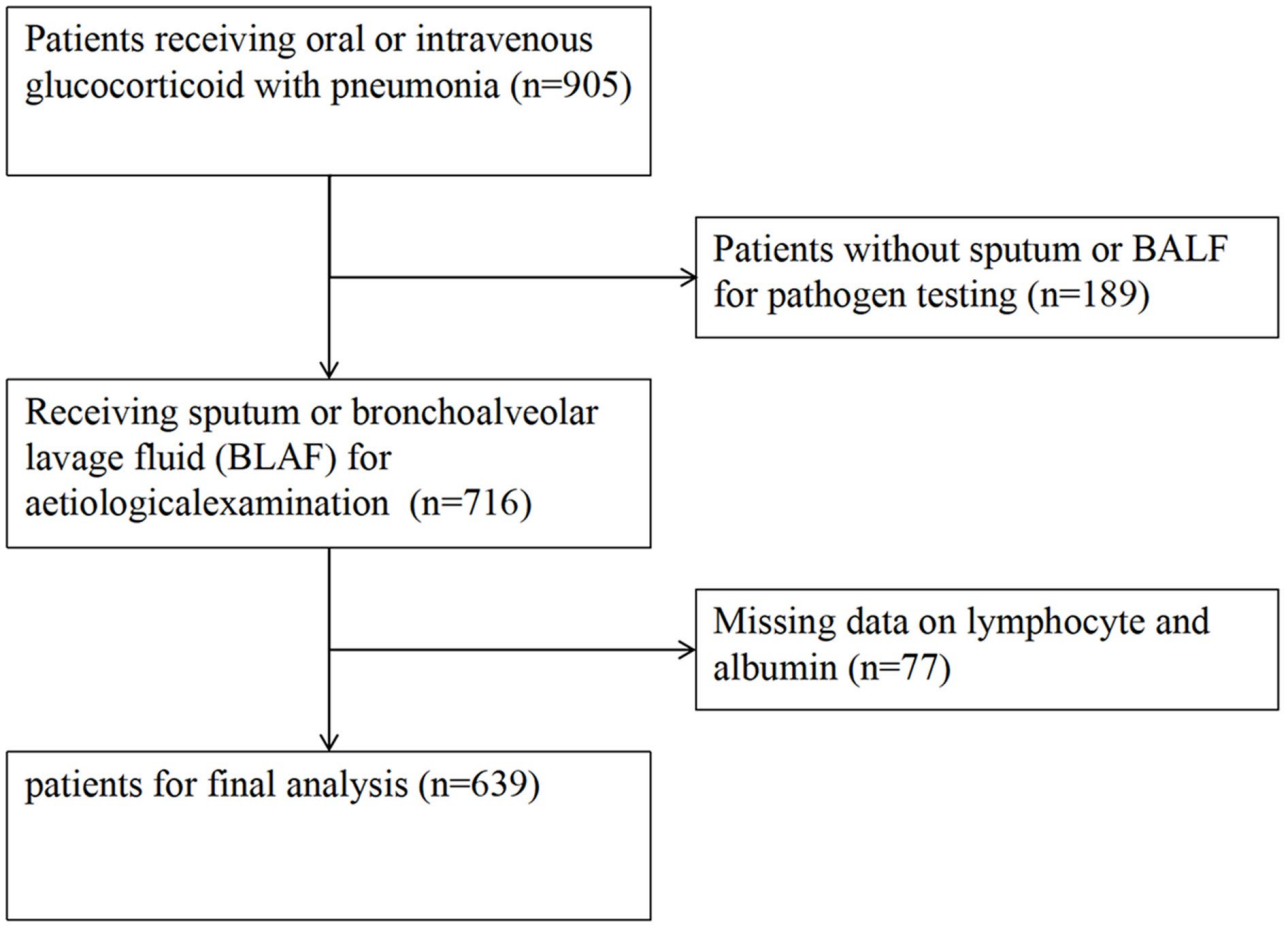
Flowchart of the study cohort.

Initially, the patient cohort consisted of 716 individuals enrolled between January 1, 2013, and December 31, 2017, from six secondary and tertiary academic hospitals across China (5). All participants had undergone oral or intravenous glucocorticoid therapy due to various clinical indications, including connective tissue disorders, chronic glomerulonephritis or nephrotic syndrome, and other clinical contexts such as malignancies, lymphoma. Pneumonia developed after a median treatment duration of 4 months (interquartile range, IQR 2, 18). Diagnosis of pneumonia strictly adhered to the guidelines (17, 18). Inclusion criteria required patients to: (1) have undergone oral or intravenous glucocorticoid therapy before hospital admission, (2) develop pneumonia either at admission or during hospitalization, and (3) be aged 16 years or older. Patients with incomplete data for peripheral blood lymphocyte counts and serum ALB concentrations were excluded (*n* = 77), leaving a final study population of 639 individuals. Figure 1 delineates the patient selection process and exclusion criteria.

### 2.3 Data extraction and PNI

The following variables were extracted for analysis: (1) demographic information including smoking habits, and alcohol intake; (2) vital signs encompassing body temperature and oxygen saturation levels; (3) comorbidities such as diabetes mellitus, nephrotic syndrome, liver cirrhosis, respiratory failure, chronic obstructive pulmonary disease (COPD) or asthma, malignancies, septic shock, and altered consciousness states; (4) laboratory parameters including blood pH, hemoglobin levels, ALB, sodium, potassium, lymphocyte count, total bilirubin, white blood cell and neutrophil counts, serum creatinine, blood urea nitrogen (BUN), platelet counts, prothrombin time, lactic acid, international normalized ratio (INR), and procalcitonin; (5) severity assessment scores based on the CURB-65 criteria; and (6) treatment interventions such as mechanical ventilation, and the cumulative glucocorticoid dosage administered.

Covariates identified as potential confounders through both literature and clinical judgment were included age, nephrotic syndrome, cirrhosis, respiratory failure, tumor, septic shock, blood urea nitrogen, serum creatinine, white blood cells, hemoglobin, INR, mechanical ventilation, glucocorticoid accumulation, vasoactive drugs, CURB-65 score (5, 11, 15). The calculation for the PNI was performed according to the following formula:

PNI = serum ALB (g/L) + 5 × lymphocyte count (10^9^/L) (19).

The primary outcomes were mortality rates at 30 days and 90 days.

### 2.4 Management of missing data

Only INR and blood oxygen saturation data had relatively high proportions of missing values (31.3%, 200/639, and 37.9%, 242/639, respectively). Missing data for other variables are summarized in Supplementary Table S1. Missing values were imputed by multiple imputation, generating ten datasets for pooled analysis.

### 2.5 Statistical analysis

Clinical characteristics and demographic data of patients were categorized based on PNI levels. Normally distributed continuous variables were summarized as means ± standard deviations (SD), whereas variables demonstrating non-normal distributions were reported as medians with corresponding interquartile ranges (IQR). Frequencies and percentages (%) were utilized to represent categorical data. To evaluate statistical differences between groups, categorical data were analyzed using Fisher’s exact tests or chi-square tests, while continuous variables were assessed using independent-sample t-tests or Mann–Whitney U tests, depending on their distribution.

The relationship between PNI values and the risk of mortality in pneumonia patients was assessed using Cox regression analysis. Kaplan–Meier analysis complemented by log-rank testing was conducted to determine variations in mortality risk predictions across different PNI categories. Previously reported studies have indicated that optimal PNI thresholds vary significantly according to specific diseases, population characteristics, and disease severity levels (20–23). Given potential nutritional and immune status variations between Chinese and Western populations, a threshold of 43 was adopted based on earlier research conducted in Guangdong, China (24), dividing patients into two distinct groups (PNI < 43 and ≥43). PNI was evaluated both categorically and continuously (with hazard ratios calculated for every two-unit decrement). Model 1 was Crude model (unadjusted). Model 2 adjusted for age, nephrotic syndrome, liver cirrhosis, respiratory failure, malignancy, and septic shock. Further covariates introduced into Model 3 included blood urea nitrogen, serum creatinine, white blood cell counts, hemoglobin concentration, and INR. Model 4 expanded upon Model 3 by incorporating additional adjustments for mechanical ventilation use, total glucocorticoid dosage, vasoactive drug administration, and CURB-65 scoring. Additionally, RCS analysis was utilized to assess and visualize the nonlinear relationships between PNI values and mortality risks.

To confirm the reliability and consistency of these results, subgroup analyses were executed, with interactions among subgroups evaluated through likelihood ratio tests. Additionally, sensitivity analyses were conducted, excluding subjects with incomplete covariate information.

All analyses were performed utilizing R (version 4.2.2) and the Free Statistics platform (version 2.1). *p* < 0.05 was established as the criterion for statistical significance.

## 3 Results

### 3.1 Patient characteristics

This study enrolled 639 patients with pneumonia who received glucocorticoid treatment, median duration of use (IQR) of glucocorticoid medication was 4 (2, 18) months. Based on their PNI values, patients were divided into two categories (< 43 and ≥ 43), and their initial clinical attributes are detailed in Table 1. In this cohort, 332 patients (52.0%) were aged 60 or older, 334 (52.3%) were male, 169 (26.4%) reported a history of smoking, and 55 (8.6%) were alcohol consumers. Mortality rates recorded at 30 days and 90 days post-admission were 22.5% (144 cases) and 26.0% (166 cases), respectively. Those with reduced PNI (< 43) demonstrated elevated body temperatures, higher incidences of nephrotic syndrome, respiratory failure, COPD, or asthma, and increased reliance on vasoactive drugs and mechanical ventilation. Additionally, lower PNI scores correlated with reduced counts of platelets, white blood cells, lymphocytes, and hemoglobin compared to patients exhibiting PNI scores ≥ 43.

**Table 1:** Characteristics of the study population (*N* = 639).

Variables	Total (*n* = 639)	PNI < 43 (*n* = 475)	PNI ≥ 43 (*n* = 164)	*p*-value
Age (year), *n* (%)				0.26
<60	307 (48.0)	222 (46.7)	85 (51.8)	
≥60	332 (52.0)	253 (53.3)	79 (48.2)	
Gender, *n* (%)				0.5
Male	334 (52.3)	252 (53.1)	82 (50)	
Female	305 (47.7)	223 (46.9)	82 (50)	
Smoke, *n* (%)				0.898
No	470 (73.6)	350 (73.7)	120 (73.2)	
Yes	169 (26.4)	125 (26.3)	44 (26.8)	
Alcoholism, *n* (%)				0.494
No	584 (91.4)	432 (90.9)	152 (92.7)	
Yes	55 (8.6)	43 (9.1)	12 (7.3)	
Temperature, Mean ± SD	37.3 ± 1.0	37.5 ± 1.1	37.0 ± 0.9	< 0.001
Heartrate, Mean ± SD	88.6 ± 24.9	89.5 ± 25.1	86.0 ± 24.4	0.114
MBP, Mean ± SD	90.6 ± 13.3	90.0 ± 13.7	92.2 ± 11.9	0.074
SPo2, Mean ± SD	94.2 ± 6.2	93.9 ± 6.5	95.0 ± 5.0	0.038
Diabetes, *n* (%)				0.032
No	473 (74.0)	362 (76.2)	111 (67.7)	
Yes	166 (26.0)	113 (23.8)	53 (32.3)	
Nephrotic syndrome, *n* (%)				< 0.001
No	562 (87.9)	405 (85.3)	157 (95.7)	
Yes	77 (12.1)	70 (14.7)	7 (4.3)	
Respiratory failure, *n* (%)				< 0.001
No	314 (49.1)	203 (42.7)	111 (67.7)	
Yes	325 (50.9)	272 (57.3)	53 (32.3)	
Tumor, *n* (%)				0.255
No	600 (93.9)	443 (93.3)	157 (95.7)	
Yes	39 (6.1)	32 (6.7)	7 (4.3)	
Septic shock, *n* (%)				0.708
No	596 (93.3)	442 (93.1)	154 (93.9)	
Yes	43 (6.7)	33 (6.9)	10 (6.1)	
Disturbance of consciousness, *n* (%)				0.067
No	606 (94.8)	446 (93.9)	160 (97.6)	
Yes	33 (5.2)	29 (6.1)	4 (2.4)	
CHD, *n* (%)				0.688
No	559 (87.5)	417 (87.8)	142 (86.6)	
Yes	80 (12.5)	58 (12.2)	22 (13.4)	
CHF, *n* (%)				0.383
No	623 (97.5)	461 (97.1)	162 (98.8)	
Yes	16 (2.5)	14 (2.9)	2 (1.2)	
Cirrhosis, *n* (%)				0.684
No	632 (98.9)	469 (98.7)	163 (99.4)	
Yes	7 (1.1)	6 (1.3)	1 (0.6)	
COPD or Asthma, *n* (%)				0.047
No	613 (95.9)	460 (96.8)	153 (93.3)	
Yes	26 (4.1)	15 (3.2)	11 (6.7)	
CRF, *n* (%)				0.946
No	593 (92.8)	441 (92.8)	152 (92.7)	
Yes	46 (7.2)	34 (7.2)	12 (7.3)	
Platelets (×109/L)	183.0 (130.0, 245.5)	174.0 (119.0, 234.5)	201.0 (158.0, 259.0)	< 0.001
WBC (×109 /L)	7.9 (5.7, 11.5)	7.8 (5.6, 11.4)	8.5 (6.4, 12.4)	0.016
Lymphocyte (×109/L)	0.8 (0.5, 1.4)	0.7 (0.4, 1.0)	1.7 (1.1, 2.2)	< 0.001
Prothrombin time (s)	13.7 (12.6, 15.9)	13.7 (12.6, 16.1)	13.5 (12.7, 15.4)	0.557
INR	1.1 (1.0, 1.4)	1.1 (1.0, 1.4)	1.1 (1.0, 1.5)	0.49
Procalcitonin (ng/mL)	0.3 (0.1, 0.8)	0.3 (0.1, 0.9)	0.3 (0.1, 0.6)	0.08
Total bilirubin (μmol/L)	9.7 (6.6, 13.6)	9.7 (6.4, 13.1)	9.7 (6.8, 14.3)	0.39
Neutrophils (×10^9^ /L)	6.5 (4.3, 10.1)	6.7 (4.3, 10.1)	6.3 (4.3, 9.9)	0.643
Serum creatinine (mmol/L)	64.0 (51.0, 89.0)	66.0 (50.2, 93.0)	62.9 (52.0, 78.8)	0.407
Lactic acid (mmol/L)	1.0 (0.0, 1.0)	1.0 (0.0, 1.0)	0.0 (0.0, 1.0)	< 0.001
PH	7.4 ± 0.1	7.4 ± 0.1	7.4 ± 0.1	0.174
Hemoglobin (g/L)	112.3 ± 23.0	110.3 ± 21.3	118.2 ± 26.6	< 0.001
Albumin (g/L)	32.8 ± 6.4	30.6 ± 4.8	39.1 ± 6.1	< 0.001
BUN (mmol/L)	6.3 (4.7, 9.8)	6.5 (4.9, 10.3)	5.8 (4.3, 8.9)	0.01
Potassium (mmol/L)	3.9 (3.6, 4.2)	3.9 (3.6, 4.2)	4.0 (3.7, 4.2)	0.328
Sodium (mmol/L)	137.7 ± 7.7	136.7 ± 5.4	140.7 ± 11.5	< 0.001
Vasoactive drugs, *n* (%)				0.003
No	529 (82.8)	381 (80.2)	148 (90.2)	
Yes	110 (17.2)	94 (19.8)	16 (9.8)	
Mechanical ventilation, *n* (%)				< 0.001
No	412 (64.5)	286 (60.2)	126 (76.8)	
Yes	227 (35.5)	189 (39.8)	38 (23.2)	
Curb-65, *n* (%)				0.065
≤ 1	455 (71.2)	329 (69.3)	126 (76.8)	
>1	184 (28.8)	146 (30.7)	38 (23.2)	
Glucocorticoid accumulation (g)	3.6 (1.9, 8.1)	3.8 (2.2, 8.7)	3.6 (1.8, 7.9)	0.31
30-day mortality, *n* (%)	144 (22.5)	127 (26.7)	17 (10.4)	< 0.001
90-day mortality, *n* (%)	166 (26.0)	144 (30.3)	22 (13.4)	< 0.001

COPD, chronic obstructive pulmonary disease; MBP, mean blood pressure; SPo2, blood oxygen saturation; BUN, blood urea nitrogen; CHD, coronary heart disease; CHF, congestive heart failure; CRF, chronic renal failure; INR, international normalized ratio; PNI, prognostic nutritional index; WBC, white blood cells.

### 3.2 Total mortality in different PNI groups

**Figure 2:**
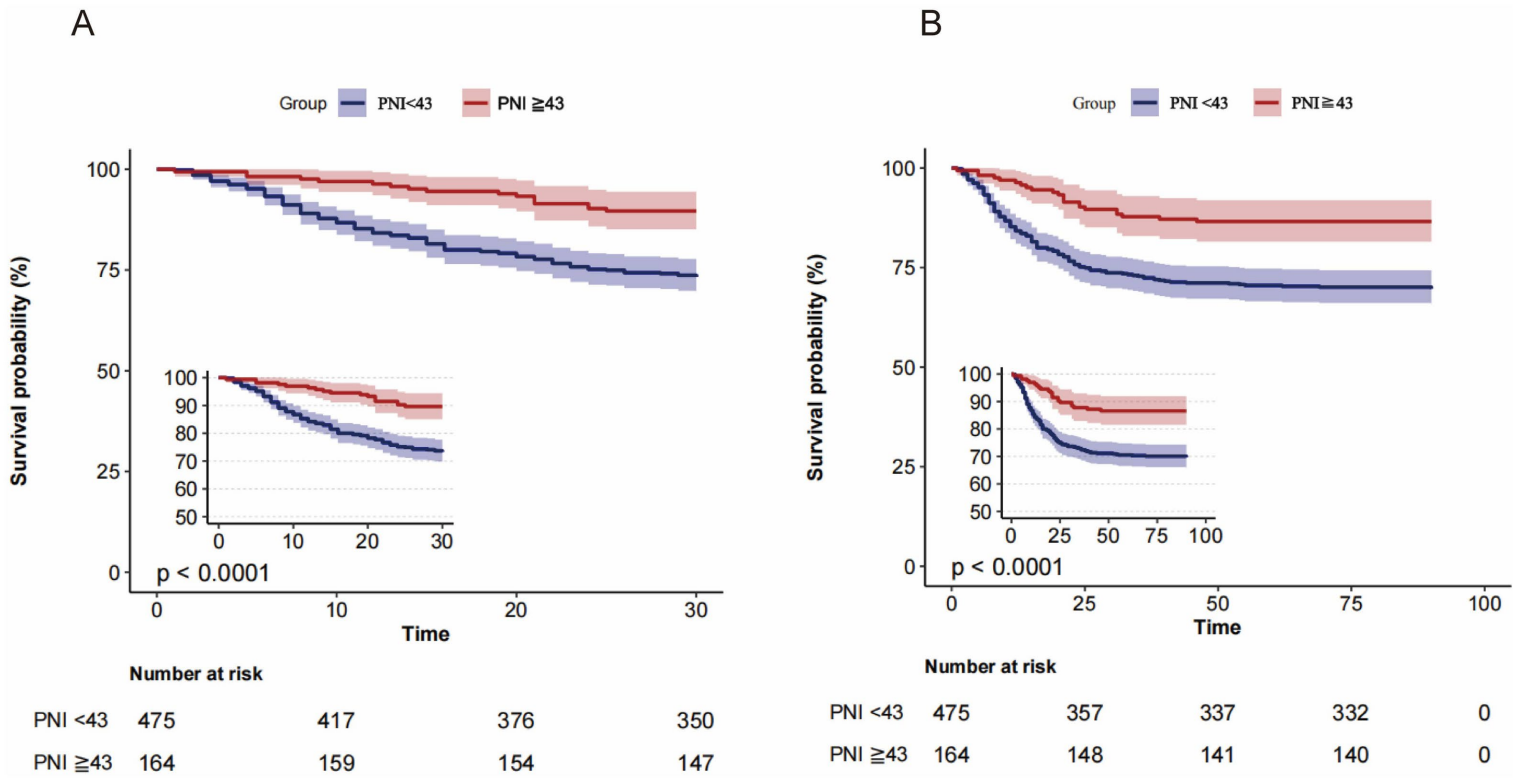
Kaplan-Meier survival curves for all-cause mortality according to the PNI dichotomous numerical classification. **(A)** 30-day and **(B)** 90-day mortality: patients with PNI < 43 had significantly lower survival compared with those with PNI ≥ 43 (*p* < 0.0001).

The 30-day mortality rate was significantly higher among patients with PNI values below 43 (26.7%) compared to those with scores of 43 or higher (10.4%). At the 90-day mark, mortality rates were similarly higher for patients with lower PNI scores (30.3%) versus those with higher scores (13.4%). As the PNI levels decreased, mortality rates increased, a trend that was further illustrated by the Kaplan–Meier survival analyses (Figure 2).

### 3.3 Relationship between PNI and 30-day mortality

Supplementary Table S2 summarizes results from the initial univariate analysis concerning 30-day mortality among pneumonia patients. Subsequent multivariate Cox regression (Table 2) revealed a robust association between decreasing PNI and higher 30-day mortality. Specifically, an unadjusted model indicated a 17% increase in mortality risk for every two-unit drop in PNI (*HR* = 1.17, 95% *CI* = 1.11–1.22, *p* < 0.001). This association persisted even after comprehensive covariate adjustments (*HR* = 1.10, 95% *CI* = 1.05–1.15, *p* < 0.001, Model 4). Categorically, patients whose PNI scores fell below 43 experienced a 188% higher mortality risk within 30 days in the crude model (*HR* = 2.88, 95% *CI* = 1.73–4.77, *p* < 0.001). After extensive adjustments, this elevated mortality risk remained significant (*HR* = 2.18, 95% *CI* = 1.28–3.81, *p* = 0.005, Model 4).

**Table 2:** Cox proportional hazard ratios (HRs) for all-cause mortality.

Variable	Model 1		Model 2		Model 3		Model 4	
	HR (95% CI)	*p*-value	HR (95% CI)	*p*-value	HR (95% CI)	*p*-value	HR (95% CI)	*p*-value
30-day mortality								
Continuous^a^	1.17 (1.11–1.22)	<0.001	1.09 (1.04–1.15)	<0.001	1.11 (1.05–1.16)	<0.001	1.10 (1.05–1.15)	<0.001
PNI ≥ 43	1 (Ref)		1 (Ref)		1 (Ref)		1 (Ref)	
PNI<43	2.88 (1.73–4.77)	<0.001	1.80 (1.08–2.99)	0.027	2.13 (1.24–3.67)	0.007	2.18 (1.28–3.81)	0.005
90-day mortality								
Continuous^a^	1.15 (1.11–1.20)	<0.001	1.08 (1.03–1.13)	0.001	1.09 (1.05–1.14)	<0.001	1.09 (1.04–1.14)	<0.001
PNI ≥ 43	1 (Ref)		1 (Ref)		1 (Ref)		1 (Ref)	
PNI<43	2.55 (1.63–3.99)	<0.001	1.59 (1.01–2.5)	0.047	1.87 (1.15–3.02)	0.012	1.96 (1.20–3.19)	0.008

^a^X was entered as a continuous variable per 2 unit decrease.

Model 1: unadjusted.

Model 2: adjusted for age, nephrotic syndrome, cirrhosis, respiratory failure, tumor, septic shock.

Model 3: adjusted for age, nephrotic syndrome, cirrhosis, respiratory failure, tumor, septic shock, blood urea nitrogen, serum creatinine, white blood cells, hemoglobin, INR.

Model 4: adjusted for age, nephrotic syndrome, cirrhosis, respiratory failure, tumor, septic shock, blood urea nitrogen, serum creatinine, white blood cells, hemoglobin, INR, mechanical ventilation, glucocorticoid accumulation, vasoactive drugs, CURB-65.

aHR, adjusted hazard ratio; CI, confidence intervals; Ref., reference; INR, international normalized ratio.

### 3.4 Association between PNI and 90-day mortality

The outcomes of the univariate regression for 90-day mortality in pneumonia patients are displayed in Supplementary Table S3. Multivariate Cox regression analyses (Table 2) consistently revealed significant correlations between lower PNI and heightened 90-day mortality risk. When analyzed as a continuous variable, the model 1 showed a 15% increased risk per 2-unit decrease in PNI (*HR* = 1.15, 95% *CI* = 1.11–1.20, *p* < 0.001). This association persisted in model 2 (*HR* = 1.08, 95% *CI* = 1.03–1.13, *p* = 0.001), model 3 (*HR* = 1.09, 95% *CI* = 1.05–1.14, *p* < 0.001) and model 4 (*HR* = 1.09, 95% *CI* = 1.04–1.14, *p* < 0.001). When PNI was used as a 2-category classification, the 90-day risk of death for patients with PNI < 43 was 2.55-fold higher than that for patients with PNI ≥ 43 (*HR* = 2.55, 95% *CI* = 1.63–3.99, *p* < 0.001, Table 2, Model 1). After adjusting for age, nephrotic syndrome, cirrhosis, respiratory failure, tumor, septic shock, blood urea nitrogen, serum creatinine, white blood cells, hemoglobin, INR, mechanical ventilation, glucocorticoid accumulation, vasoactive medications and CURB-65, this association remained signifacant (*HR* = 1.96, 95% *CI* = 1.20–3.19, *p* = 0.008, Table 2, model 4).

### 3.5 RCS analysis of PNI and ACM

Fully adjusted RCS analysis supported a linear association between PNI values and mortality outcomes at both 30 and 90 days (Supplementary Figure S1).

### 3.6 Subgroup analysis

**Figure 3:**
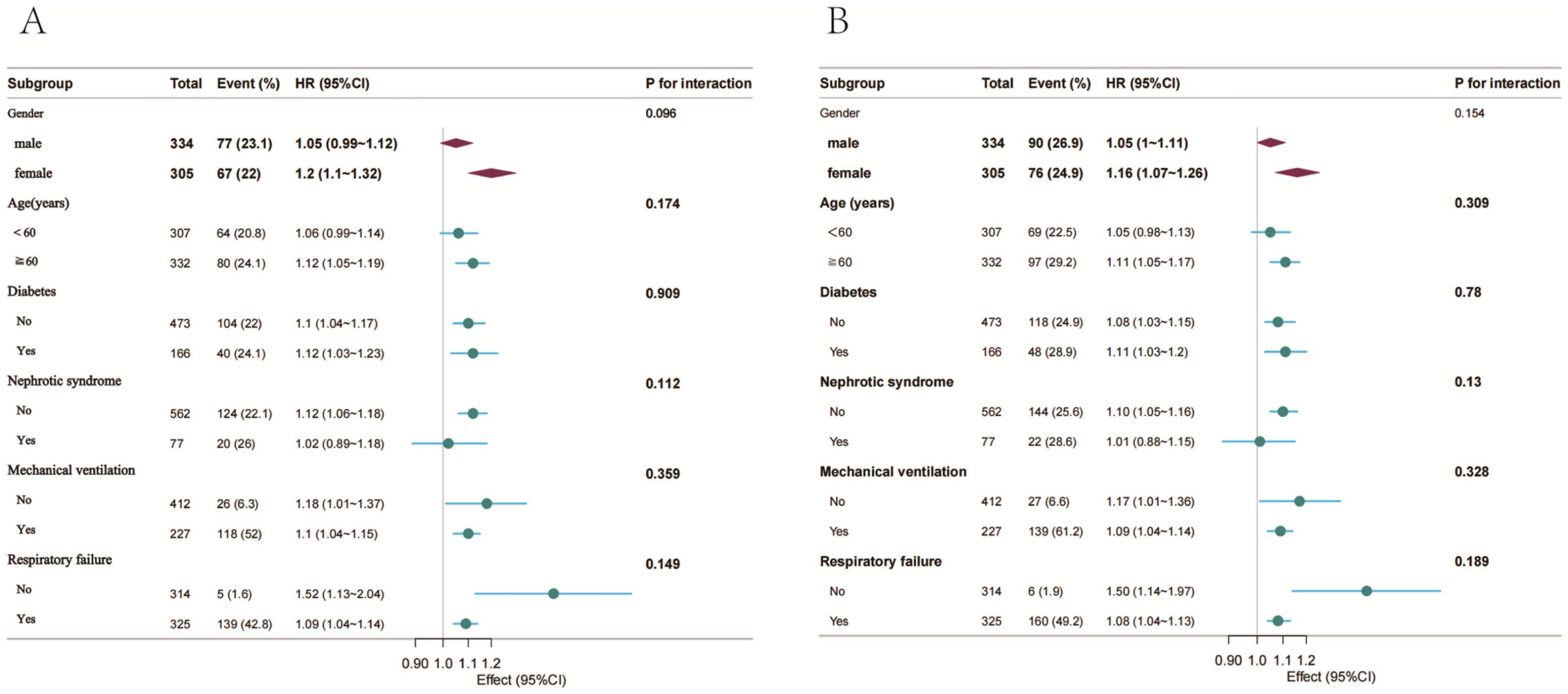
Forest plot of HRs for the 30-day and 90-day mortality in different subgroups. **(A)** 30-day mortality; **(B)** 90-day mortality; Adjusted for age, nephrotic syndrome, cirrhosis, respiratory failure, tumor, septic shock, blood urea nitrogen, serum creatinine, white blood cells, hemoglobin, INR, mechanical ventilation, glucocorticoid accumulation, vasoactive drugs, CURB-65.

Subgroup evaluations and interaction analyses assessed the consistency of the association between PNI and mortality across various demographic and clinical subpopulations (Figure 3). Consistent inverse associations between PNI values and mortality at both 30 and 90 days were evident across all examined subgroups, with no significant subgroup interactions observed (*p* > 0.05).

### 3.7 Sensitivity analysis

In sensitivity analyses, after omitting individuals with incomplete covariate data, the associations between PNI and mortality were stable among 358 pneumonia patients receiving glucocorticoids. Each two-unit reduction in PNI corresponded with a 10% increase in mortality risk at both 30 and 90 days in fully adjusted models. Crude analyses revealed that patients with PNI scores below 43 had a 244% higher risk of 30-day mortality (*p* = 0.001, Supplementary Table S4, Model 1) and a 210% higher risk of 90-day mortality (*p* = 0.001, Supplementary Table S5, Model 1) compared to those with PNI ≥ 43. After full adjustments, significantly elevated mortality risks remained, showing a 136% higher risk at 30 days (*HR* = 2.36, 95% *CI* = 1.08–5.18, *p* = 0.031, Supplementary Table S4, Model 4) and a 117% higher risk at 90 days (*HR* = 2.17, 95% *CI* = 1.07–4.41, *p* = 0.031, Supplementary Table S5, Model 4).

## 4 Discussion

Prolonged administration of corticosteroids at high doses can significantly suppress immune function, elevating susceptibility to severe infections. Among immunocompromised individuals, pulmonary infections attributable to corticosteroid-induced immune suppression constitute a leading cause of death (5, 25), placing substantial strain on healthcare systems. Currently, there is a scarcity of effective biomarkers for predicting these outcomes. Within our study involving 639 participants, the mortality rates recorded at 30 and 90 days were 22.5 and 26.0%, respectively. Following rigorous adjustment for potential confounders, every two-unit drop in PNI was associated with an increased risk of mortality, 10% at the 30-day checkpoint and 9% at the 90-day checkpoint. Patients with PNI levels less than 43 exhibited substantially elevated risks of mortality, with increases of 118% at 30 days and 96% at 90 days.

Prior study’s have similarly explored the correlation between PNI and clinical outcomes among pneumonia patients. For example, Lisa and colleagues reported a 13.6% increase in mortality risk per unit decrease in PNI for individuals diagnosed with community-acquired bacterial pneumonia, even after adjustments for the Charlson Comorbidity Index and patient age (26). In contrast, our findings indicated a relatively lower risk, possibly attributable to our cohort’s higher median PNI values (median [IQR]: 37.9 [32.1, 43.2]), whereas Lisa et al. reported lower mean PNI scores (survivors: 34.7 ± 4.5, non-survivors: 30.1 ± 6.5). Another previous study (15) confirmed an inverse relationship between PNI and mortality at 30 and 90 days among CAP patients, although their study predominantly involved ICU admissions. Our research specifically targeted hospitalized pneumonia patients who received glucocorticoids either alone or combined with other immunosuppressive therapies. This is a crucial differentiation since prolonged glucocorticoids therapy uniquely increases vulnerability to immunosuppression and severe infectious complications (27). Furthermore, a study utilizing data from the MIMIC-III database indicated that critically ill patients with PNI below 35.07 had increased mortality risks of 21.6 and 23.3% at 30 and 90 days, respectively, compared to those with higher PNI (28).

Furthermore, diminished PNI values have consistently been linked with higher mortality risks in COVID-19 pneumonia cohorts (29–32). Park et al. (33) highlighted the prognostic role of PNI in lung cancer patients undergoing surgery, reporting elevated postoperative complications—including delirium and pneumonia—in those with a preoperative PNI under 50. In contrast, Shimoyama et al., studying a smaller cohort (*n* = 33), did not detect significant variations in PNI between pneumonia survivors and non-survivors (34), a discrepancy potentially attributable to their limited sample size. Collectively, this evidence supports the potential application of PNI as a reliable biomarker for evaluating nutritional health, facilitating timely interventions, and improving patient stratification in respiratory diseases.

Derived from lymphocyte counts and serum ALB measurements, PNI effectively reflects overall nutritional health, inflammatory conditions, and immune response, thus functioning as an important prognostic tool for patient outcomes (35). These factors critically influence the onset and progression of pneumonia (36–38). Prolonged or high-dose glucocorticoid treatment adversely impacts serum ALB concentration and lymphocyte counts, thereby compromising patient outcomes.

Serum ALB, synthesized exclusively by the liver, is essential for maintaining plasma colloid osmotic pressure, transporting various metabolites, and supporting nutritional status (39). Glucocorticoids enhance protein breakdown through mechanisms involving ubiquitin-proteasome and autophagy-lysosomal pathways (40, 41). Concurrently, they hinder protein synthesis by blocking eukaryotic initiation factor 4E-binding protein 1 and ribosomal protein S6 kinase beta-1 (41). Glucocorticoid usage also predisposes individuals to gastrointestinal complications such as gastritis and ulcers, potentially impairing nutrient absorption and subsequently causing hypoproteinemia (42, 43). The reported prevalence of malnutrition among pneumonia patients is highly variable, ranging from 39.4% in community-acquired pneumonia cases (44) to around 71.8% among COVID-19 patients (45). Such nutritional deficits strongly correlate with negative clinical outcomes, including prolonged hospitalizations, increased mortality, and frequent ICU admissions (46).

Inflammation and nutrition exhibit an interdependent relationship, where inflammation may trigger insulin resistance and catabolism, thus exacerbating malnutrition (47). Conversely, nutritional deficits can result in mitochondrial dysfunction, elevated reactive oxygen species generation, and inflammatory pathway activation (48).

Peripheral lymphocytes, encompassing T cells, B cells, and natural killer cells, constitute key immune system components. Glucocorticoids inhibit lymphocyte proliferation and diminish immune functionality, thereby reducing lymphocyte counts and impairing overall immune response efficacy (49). Reduced CD4+, CD8+, and natural killer cells correlate with increased mortality in pneumocystis pneumonia among HIV-negative individuals (50). Lower absolute lymphocyte counts are also associated with increased pneumonia risk in lung transplant recipients, especially when using triple immunosuppression (51). Studies show that even a low-normal lymphocyte count (1–2 × 10^9^cells/L) is associated with higher mortality at 28 days and 1 year (52). Furthermore, following pathogen invasion, neutrophils eliminate the invading pathogens through phagocytosis and release substantial quantities of cytokines possessing antimicrobial activity. However, excessive neutrophil activation can initiate a pathological cascade through the release of cytotoxic mediators, such as proteases and reactive oxygen species. This process concurrently recruits other immune cells, including lymphocytes, monocytes, and natural killer cells, disrupting the body’s anti-inflammatory homeostasis. The resultant amplification of damage-associated responses compromises overall immune function, culminating in clinical deterioration in patients with pneumonia (53).

PNI reflects the patient’s immunonutritional state. For individuals with chronic underlying conditions receiving long-term glucocorticoids prior to pneumonia, an integrated multidisciplinary approach is necessary (54). Maintaining respiratory and hemodynamic stability and promptly assessing PNI to evaluate immunonutritional status are recommended (55). When PNI is <43, intensified nutritional support should begin as outlined by Zhu et al. (56): (1) Establish a dedicated nutritional intervention team comprising attending physicians or higher-level clinicians, clinical dietitians, and specialized nurses. (2) Develop a personalized dietary plan based on the patient’s dietary habits and nutritional requirements. This plan should specify food types, quantities, and implement a combined enteral and parenteral nutritional strategy. Concurrently, administer intravenous supplements including water-soluble vitamins, compound amino acid injections, and lipid emulsion injections. (3) Conduct reassessments every 3 days thereafter, adjusting the nutritional intervention protocol promptly based on the evaluation results. (4) Following discharge, provide dietary guidance via platforms such as WeChat or telephone consultations. However, the timing of PNI assessment should be individualized, weighing both clinical status and socioeconomic factors.

Nevertheless, several limitations must be considered in interpreting these findings. Firstly, given the observational study design, it was impossible to conclusively establish a causal association between PNI values and pneumonia-related mortality. Secondly, our relatively limited sample size restricted our ability to comprehensively examine the potential interactions between multiple clinical variables. Thirdly, although we extensively controlled for known confounding factors in our multivariate models, there remains a possibility of residual confounding from unrecorded variables such as C-reactive protein, interleukin-6, procalcitonin levels, ALB infusion, or nutritional interventions, which might affect the observed PNI-mortality relationship. It is known that systemic inflammation elevates capillary permeability and hampers lymphocyte proliferation (57), thereby decreasing PNI and establishing a direct inflammation-PNI link. Furthermore, elevated CRP and IL-6 concentrations are known independent predictors of mortality linked to cardiovascular complications and infections, potentially overstating the association between PNI and mortality risk (58). Therefore, careful interpretation of these results is necessary. Subsequent investigations should consider including additional inflammatory biomarkers, such as IL-6 and CRP, to clarify how PNI influences clinical prognosis, determine whether PNI acts as an intermediary linking inflammation to clinical outcomes, and precisely measure the magnitude of any mediation effect. Furthermore, the absence of comprehensive information regarding glucocorticoid dosing protocols and delivery routes (intravenous, oral, or mixed regimens) restricted our ability to accurately evaluate the degree of immunosuppressive effect. Nonetheless, given the relative homogeneity of our patient group and thorough adjustments for critical confounding variables, the lack of granular glucocorticoid treatment details is unlikely to materially affect the validity of our conclusions. This research lays important foundations for future work designed to further confirm the prognostic significance of PNI across broader and diverse patient groups. Future studies should independently validate these findings by leveraging alternate databases or conducting prospective cohort analyses, thereby strengthening confidence in their applicability and robustness.

## 5 Conclusion

In pneumonia patients receiving glucocorticoid therapy, PNI was inversely associated with 30-day and 90-day mortality rates. Clinicians should carefully monitor pneumonia patients with reduced PNI who are undergoing glucocorticoid treatment, in order to provide optimal care and improve patient prognosis.

## Data Availability

The datasets presented in this study can be found in online repositories. The names of the repository/repositories and accession number(s) can be found below: the datasets analysed during the current study are available in the Dryad database repository, https://datadryad.org/dataset/doi:10.5061/dryad.mkkwh70x2.

## Footnotes

^1^ https://datadryad.org/stash

## Supplementary material

SUPPLEMENTARY FIGURE S1 The linear relationship between PNI and all-cause mortality. **(A)** Fit curves for 30-day mortality, **(B)** fit curves for 90-day mortality. Solid and dashed lines represent the predicted value and 95% confidence intervals. Adjusted for age, nephrotic syndrome, cirrhosis, respiratory failure, tumor, septic shock, blood urea nitrogen, serum creatinine, white blood cells, hemoglobin, INR, mechanical ventilation, glucocorticoid accumulation, vasoactive drugs, CURB-65. Only 99.5% of the data is shown.
