# Supplementary figures and images for: The impact of decreased prognostic nutritional index on the prognosis of patients with pneumonia treated with glucocorticoids: a multicenter retrospective cohort study
Source: Front Nutr. 2025 Sep 15;12:1625531. doi: 10.3389/fnut.2025.1625531 (PMC12477016; doi:10.3389/fnut.2025.1625531)

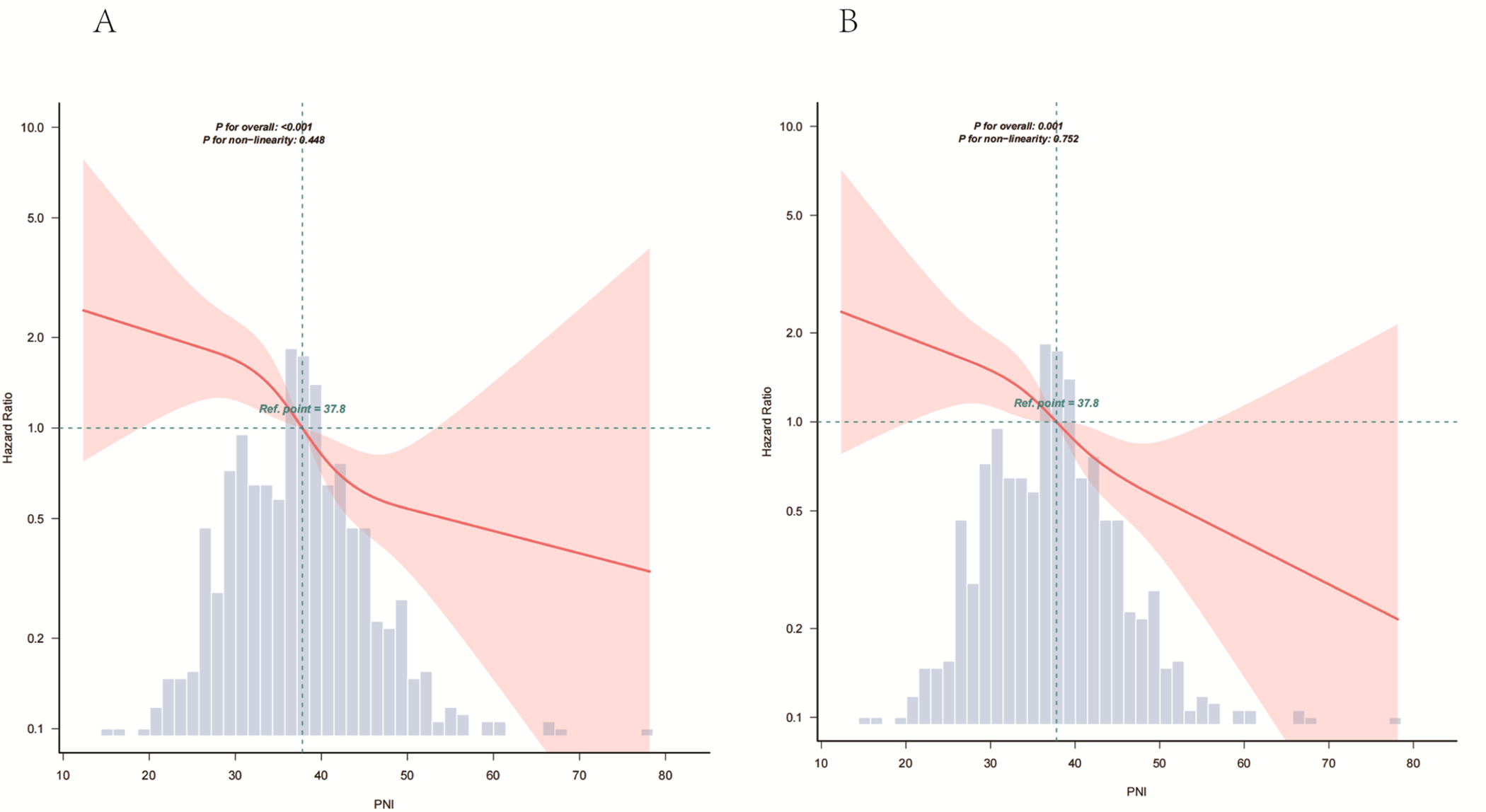

Supplement: SUPPLEMENTARY FIGURE S1 — The linear relationship between PNI and all-cause mortality. (A) Fit curves for 30-day mortality, (B) fit curves for 90-day mortality. Solid and dashed lines represent the predicted value and 95% confidence intervals. Adjusted for age, nephrotic syndrome, cirrhosis, respiratory failure, tumor, septic shock, blood urea nitrogen, serum creatinine, white blood cells, hemoglobin, INR, mechanical ventilation, glucocorticoid accumulation, vasoactive drugs, CURB-65. Only 99.5% of the data is shown. [file Image_1.tif]
