# Supplementary material for: The impact of decreased prognostic nutritional index on the prognosis of patients with pneumonia treated with glucocorticoids: a multicenter retrospective cohort study
Source: Front Nutr. 2025 Sep 15;12:1625531. doi: 10.3389/fnut.2025.1625531 (PMC12477016; doi:10.3389/fnut.2025.1625531)
Supplement: Supplementary file 2 [file Table_1.docx]

Supplementary Table S1 details of missing data

| Variable | Miss.freq | Miss.percentage% |
| --- | --- | --- |
| Age | 0 | 0 |
| Gender | 0 | 0 |
| Smoke | 0 | 0 |
| Alcoholism | 0 | 0 |
| Heartrate | 0 | 0 |
| MBP | 0 | 0 |
| SPo2 | 242 | 37.8717 |
| Temperature | 34 | 5.3208 |
| Albumin | 0 | 0 |
| BUN | 6 | 0.939 |
| Serum creatinine | 5 | 0.7825 |
| Potassium | 6 | 0.939 |
| Sodium | 10 | 1.5649 |
| CHD | 0 | 0 |
| CHF | 0 | 0 |
| Cirrhosis | 0 | 0 |
| COPD or Asthma | 0 | 0 |
| CRF | 0 | 0 |
| Curb-65 | 0 | 0 |
| Disturbance of consciousness | 0 | 0 |
| Diabetes | 0 | 0 |
| Glucocorticoid accumulation | 76 | 11.8936 |
| Hemoglobin | 0 | 0 |
| INR | 200 | 31.2989 |
| Lactic acid | 14 | 2.1909 |
| Lymphocyte | 0 | 0 |
| Nephrotic syndrome | 0 | 0 |
| Neutrophils | 0 | 0 |
| Procalcitonin | 152 | 23.7872 |
| PH | 122 | 19.0923 |
| Platelets | 11 | 1.7214 |
| PNI | 0 | 0 |
| prothrombin time | 190 | 29.734 |
| Respiratory failure | 0 | 0 |
| Septic shock | 21 | 3.2864 |
| Total bilirubin | 152 | 23.7872 |
| Tumor | 0 | 0 |
| Vasoactive drugs | 1 | 0.1565 |
| Ventilation | 0 | 0 |
| WBC | 0 | 0 |
| 30-day mortality | 0 | 0 |
| 90-day mortality | 0 | 0 |

Abbreviations: COPD, chronic obstructive pulmonary disease; MBP, mean blood pressure; SPo2, blood oxygen saturation; BUN, blood urea nitrogen; CHD, coronary heart disease; CHF, congestive heart failure; CRF, chronic renal failure; INR, international normalized ratio; PNI, prognostic nutritional index; WBC, white blood cells.
