# Supplementary material for: The impact of decreased prognostic nutritional index on the prognosis of patients with pneumonia treated with glucocorticoids: a multicenter retrospective cohort study
Source: Front Nutr. 2025 Sep 15;12:1625531. doi: 10.3389/fnut.2025.1625531 (PMC12477016; doi:10.3389/fnut.2025.1625531)
Supplement: Supplementary file 3 [file Table_2.docx]

Supplementary Table S2 Univariate regression analysis of 30-day mortality in pneumonia patients receiving glucocorticoids

| Item | HR (95% CI) | *P*-value |
| --- | --- | --- |
| Age: ≥ 60 vs ＜60 | 1.16 (0.84,1.62) | 0.365 |
| Gender: female vs male | 0.96 (0.69,1.33) | 0.808 |
| Alcoholism: Yes vs No | 1.43 (0.85,2.4) | 0.18 |
| Temperature (℃) | 1.32 (1.14,1.52) | < 0.001 |
| Heartrate (bpm) | 1.0085 (1.001,1.0159) | 0.026 |
| MBP (mmHg) | 0.9971 (0.9848,1.0096) | 0.651 |
| SPo2 | 0.95 (0.94,0.97) | < 0.001 |
| CHD: Yes vs No | 1.01 (0.62,1.66) | 0.964 |
| CHF: Yes vs No | 0.77 (0.25,2.41) | 0.653 |
| Diabetes: Yes vs No | 1.1 (0.76,1.58) | 0.621 |
| CRF: Yes vs No | 0.94 (0.49,1.79) | 0.85 |
| Nephrotic syndrome: Yes vs No | 1.24 (0.78,2) | 0.365 |
| Cirrhosis: Yes vs No | 0.63 (0.09,4.5) | 0.645 |
| Respiratory failure: Yes vs No | 34.86 (14.28,85.1) | < 0.001 |
| COPD or Asthma: Yes vs No | 0.65 (0.24,1.77) | 0.402 |
| Tumor: Yes vs No | 1.18 (0.62,2.24) | 0.619 |
| Septic shock: Yes vs No | 0.67 (0.32,1.44) | 0.31 |
| Disturbance of consciousness: Yes vs No | 3.34 (2.01,5.55) | < 0.001 |
| PH | 0.03 (0.01,0.12) | < 0.001 |
| Haemoglobin (g/L) | 0.9937 (0.987,1.0004) | 0.065 |
| Albumin (g/L) | 0.92 (0.89,0.94) | < 0.001 |
| Sodium (mmol/L) | 0.95 (0.92,0.98) | 0.002 |
| Platelets (×109/L ) | 0.996 (0.994,0.998) | < 0.001 |
| Vasoactivedrugs:Yes vs No | 8.5 (6.11,11.84) | < 0.001 |
| Ventilation: Yes vs No | 11.09 (7.25,16.98) | < 0.001 |
| PNI^a^ | 1.17 (1.11,1.22) | < 0.001 |
| Smoke:Yes vs No | 1.19 (0.83,1.7) | 0.346 |
| Curb-65: > 1 vs ≤1 | 2.6 (1.88,3.61) | < 0.001 |
| WBC (×109 /L) | 1.05 (1.03,1.07) | < 0.001 |
| Platelets (×109/L ) | 0.996 (0.994,0.998) | < 0.001 |
| Potassium (mmol/L) | 1.04 (1.02,1.05) | < 0.001 |
| Lymphocyte (×109/L ) | 0.65 (0.5,0.85) | 0.002 |
| Total bilirubin (μmol/L) | 1.0039 (1.0001,1.0076) | 0.045 |
| Neutrophils (×10^9^ /L) | 1.02 (1.01,1.03) | < 0.001 |
| BUN (mmol/L) | 1.04 (1.03,1.06) | < 0.001 |
| Glucocorticoid accumulation (g) | 0.99 (0.97,1) | 0.128 |
| Prothrombin time (s) | 0.9945 (0.9885,1.0006) | 0.077 |
| INR | 0.9994 (0.9774,1.0218) | 0.957 |
| Procalcitonin (ng/mL) | 1.01 (1,1.02) | < 0.001 |
| Serum creatinine (mmol/L) | 1.0013 (1,1.0026) | 0.048 |
| Lactic acid (mmol/L) | 1.21 (1.14,1.3) | < 0.001 |

^a^ X was entered as a continuous variable per 2 unit decrease.

Abbreviations: COPD, chronic obstructive pulmonary disease; MBP, mean blood pressure; SPo2, blood oxygen saturation; BUN, blood urea nitrogen; CHD, coronary heart disease; CHF, congestive heart failure; CRF, chronic renal failure; INR, international normalized ratio; PNI, prognostic nutritional index; WBC, white blood cells.
