# Supplementary material for: The impact of decreased prognostic nutritional index on the prognosis of patients with pneumonia treated with glucocorticoids: a multicenter retrospective cohort study
Source: Front Nutr. 2025 Sep 15;12:1625531. doi: 10.3389/fnut.2025.1625531 (PMC12477016; doi:10.3389/fnut.2025.1625531)
Supplement: Supplementary file 4 [file Table_3.docx]

Supplementary Table S3 Univariate regression analysis of 90-day mortality in pneumonia patients receiving glucocorticoids

| Item | HR (95% CI) | *P*-value |
| --- | --- | --- |
| Age: ≥ 60 vs ＜60 | 1.32 (0.97,1.8) | 0.075 |
| Gender: female vs male | 0.93 (0.68,1.26) | 0.637 |
| Alcoholism: Yes vs No | 1.3 (0.79,2.15) | 0.302 |
| Temperature (℃) | 1.32 (1.16,1.51) | < 0.001 |
| Heartrate (bpm) | 1.0055 (0.9988,1.0123) | 0.11 |
| MBP (mmHg) | 0.996 (0.9845,1.0075) | 0.493 |
| SPo2 | 0.95 (0.94,0.97) | < 0.001 |
| CHD: Yes vs No | 1.14 (0.73,1.77) | 0.558 |
| CHF: Yes vs No | 1.37 (0.61,3.09) | 0.451 |
| Diabetes: Yes vs No | 1.17 (0.84,1.64) | 0.361 |
| CRF: Yes vs No | 0.98 (0.55,1.77) | 0.95 |
| Nephrotic syndrome: Yes vs No | 1.18 (0.75,1.84) | 0.475 |
| Cirrhosis:Yes vs No | 0.54 (0.08,3.83) | 0.535 |
| Respiratory failure: Yes vs No | 35.27 (15.6,79.73) | < 0.001 |
| COPD or Asthma:Yes vs No | 0.7 (0.29,1.71) | 0.439 |
| Tumor: Yes vs No | 1.12 (0.61,2.07) | 0.708 |
| Septic shock: Yes vs No | 0.76 (0.39,1.48) | 0.414 |
| Disturbance of consciousness: Yes vs No | 3.1 (1.9,5.06) | < 0.001 |
| PH | 0.03 (0.01,0.12) | < 0.001 |
| Haemoglobin (g/L) | 0.993 (0.9868,0.9992) | 0.028 |
| Albumin (g/L) | 0.92 (0.9,0.94) | < 0.001 |
| Sodium (mmol/L) | 0.95 (0.92,0.98) | < 0.001 |
| Platelets (×109/L ) | 0.9961 (0.9943,0.998) | < 0.001 |
| Vasoactive drugs: Yes vs No | 9.88 (7.23,13.5) | < 0.001 |
| Ventilation: Yes vs No | 13.57 (8.97,20.52) | < 0.001 |
| PNI^a^ | 1.15 (1.11,1.2) | < 0.001 |
| smoke: Yes vs No | 1.18 (0.84,1.64) | 0.342 |
| Curb-65: >1 vs ≤1 | 2.84 (2.09,3.85) | < 0.001 |
| WBC (×109 /L) | 1.05 (1.03,1.07) | < 0.001 |
| Platelets (×109/L ) | 0.9961 (0.9943,0.998) | < 0.001 |
| Potassium (mmol/L) | 1.04 (1.02,1.05) | < 0.001 |
| Lymphocyte (×109/L ) | 0.66 (0.52,0.85) | 0.001 |
| Total bilirubin (μmol/L) | 1.0037 (1.0001,1.0073) | 0.044 |
| Neutrophils (×10^9^ /L) | 1.02 (1.01,1.03) | < 0.001 |
| BUN (mmol/L) | 1.05 (1.03,1.06) | < 0.001 |
| Glucocorticoid accumulation (g) | 0.98 (0.97,1) | 0.042 |
| Prothrombin time (s) | 0.9944 (0.9888,1.0001) | 0.052 |
| INR | 0.9972 (0.9749,1.0201) | 0.811 |
| Procalcitonin (ng/mL) | 1.0093 (1.0035,1.0152) | 0.002 |
| Serum creatinine (mmol/L) | 1.0011 (0.9998,1.0024) | 0.097 |
| Lactic acid (mmol/L) | 1.21 (1.14,1.29) | < 0.001 |

^a^ X was entered as a continuous variable per 2 unit decrease.

Abbreviations: COPD, chronic obstructive pulmonary disease; MBP, mean blood pressure; SPo2, blood oxygen saturation; BUN, blood urea nitrogen; CHD, coronary heart disease; CHF, congestive heart failure; CRF, chronic renal failure; INR, international normalized ratio; PNI, prognostic nutritional index; WBC, white blood cells.
