# Supplementary material for: The impact of decreased prognostic nutritional index on the prognosis of patients with pneumonia treated with glucocorticoids: a multicenter retrospective cohort study
Source: Front Nutr. 2025 Sep 15;12:1625531. doi: 10.3389/fnut.2025.1625531 (PMC12477016; doi:10.3389/fnut.2025.1625531)
Supplement: Supplementary file 6 [file Table_5.docx]

Supplementary Table S5 Cox proportional hazard ratios (HRs) for 90 days all-cause mortality

| Variable | No | n.event_% | Model 1 | | Model 2 | | Model 3 | | Model 4 | |
| --- | --- | --- | --- | --- | --- | --- | --- | --- | --- | --- |
|  |  |  | HR (95%CI) | *P* value | HR (95%CI) | *P* value | HR (95%CI) | *P* value | HR (95%CI) | *P* value |
| continuous^a^ | 358 | 100 (27.9) | 1.17 (1.11-1.24) | <0.001 | 1.12 (1.05-1.19) | <0.001 | 1.14 (1.07-1.22) | <0.001 | 1.10 (1.03-1.18) | 0.003 |
| PNI ≥ 43 | 83 | 10 (12) | 1(Ref) |  | 1(Ref) |  | 1(Ref) |  | 1(Ref) |  |
| PNI＜43 | 275 | 90 (32.7) | 3.10 (1.61-5.96) | 0.001 | 2.12 (1.09-4.11) | 0.026 | 2.47 (1.24-4.93) | 0.01 | 2.17 (1.07-4..41) | 0.031 |

^a^ X was entered as a continuous variable per 2 unit decrease.

Model 1:unadjusted.

Model 2: adjusted for age, nephrotic syndrome, cirrhosis, respiratory failure, tumor, septic shock.

Model 3: adjusted for age, nephrotic syndrome, cirrhosis, respiratory failure, tumor, septic shock, blood urea nitrogen, serum creatinine, white blood cells, hemoglobin, INR.

Model 4: adjusted for age, nephrotic syndrome, cirrhosis, respiratory failure, tumor, septic shock, blood urea nitrogen, serum creatinine, white blood cells, hemoglobin, INR, mechanical ventilation, glucocorticoid accumulation, vasoactive drugs, Curb-65.

Abbreviations: aHR, adjusted hazard ratio; CI, confidence intervals; Ref., reference; INR, international normalized ratio.
